# Editorial: Intervention effects of food-derived polyphenols and bioactive peptides on chronic inflammation

**DOI:** 10.3389/fnut.2024.1493706

**Published:** 2024-09-19

**Authors:** Jiaojiao Zhang, Danila Cianciosi, Md Obaidul Islam, Lei Zhang

**Affiliations:** ^1^College of Food and Health, Zhejiang A&F University, Hangzhou, China; ^2^Department of Clinical Sciences, Faculty of Medicine, Polytechnic University of Marche, Ancona, Italy; ^3^UHealth IT, University of Miami, Miami, FL, United States; ^4^Department of Chemical Engineering, Waterloo Institute for Nanotechnology, University of Waterloo, Waterloo, ON, Canada

**Keywords:** food-derived, polyphenol, bioactive, peptide, chronic inflammation

## Introduction

Chronic inflammation is a pivotal factor in the development of numerous chronic diseases (1), including cardiovascular diseases, neurodegenerative disorders, anemia of inflammation, metabolic syndromes and even cancer (2, 3). Chronic inflammation is even considered the underlying cause of diseases throughout the lifespan (4). The critical link between nutrition and immunity, particularly in the context of chronic illnesses, highlights the importance of diet in controlling chronic inflammation. Micronutrient deficiencies are key drivers of inflammation and are closely associated with increased morbidity and mortality, emphasizing the need for proper nutrient intake to manage inflammatory responses (5). Proper nutritional support, including the intake of anti-inflammatory nutrients, plays a pivotal role in mitigating the effects of chronic inflammation and reducing disease progression. This underscores the importance of exploring dietary interventions that can actively modulate inflammatory pathways and improve health outcomes.

**Figure 1:**
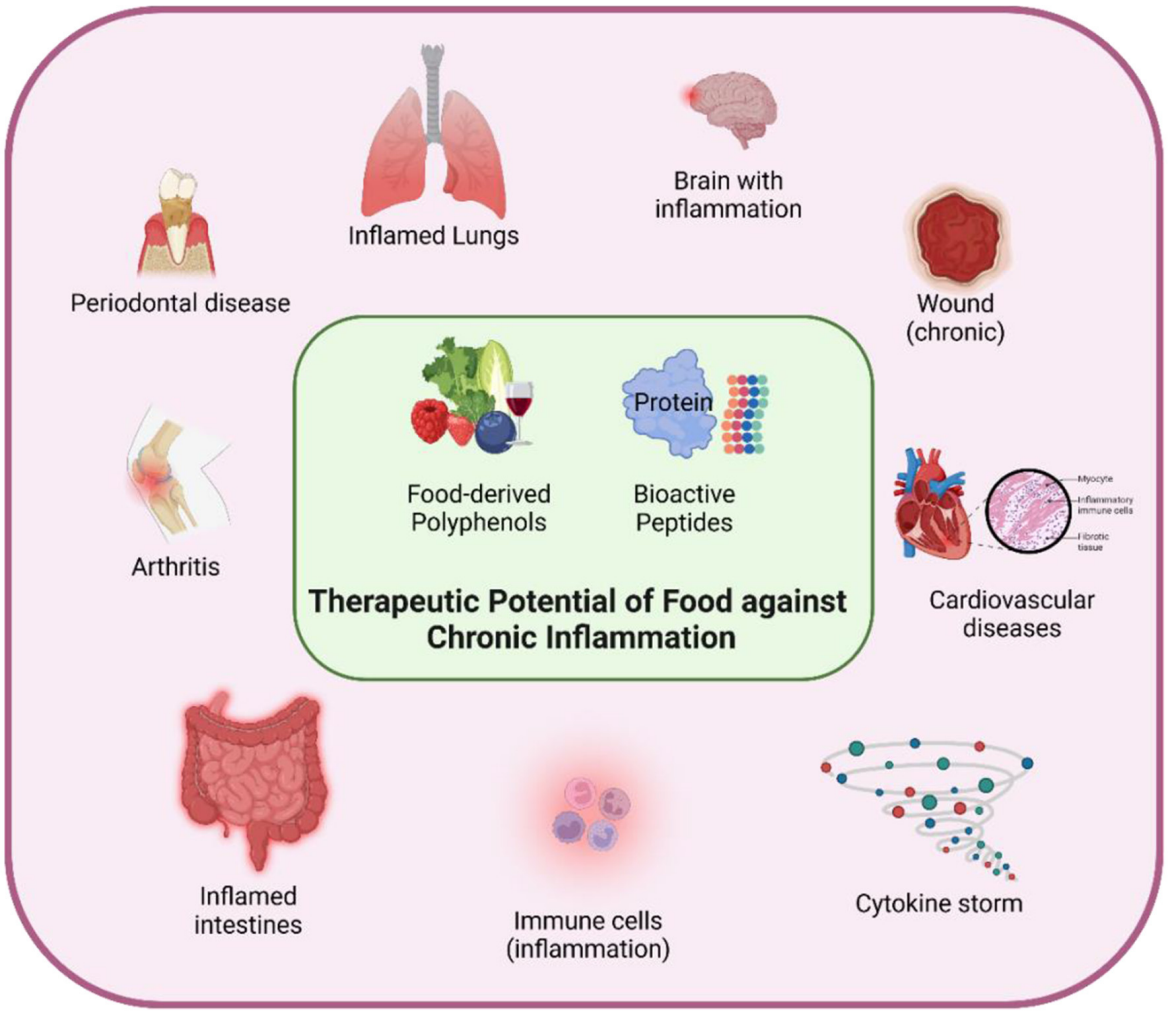
The figure illustrates the synergistic potential of food-derived polyphenols and bioactive peptides in mitigating chronic inflammation. The collective manuscripts suggest that incorporating these compounds into the diet could provide a therapeutic strategy for managing chronic inflammatory conditions.

Hence, the growing interest in food-derived polyphenols and bioactive peptides as potential therapeutic agents (Figure 1) for managing chronic inflammation has inspired extensive research into their molecular mechanisms and practical applications (6–8). However, the therapeutic use of polyphenols is often hampered by their poor stability and bioavailability. Nanotechnology-based delivery systems have shown promise in addressing these limitations by improving the stability, bioactivity, bioavailability, and cellular uptake of these compounds (9).

The complex interplay among diet, inflammation, and chronic diseases has emerged as a key focus in nutritional research. This Research Topic, shared across *Frontiers in Nutrition, Frontiers in Immunology*, and *Frontiers in Chemistry*—particularly within the *Nutritional Immunology* and *Food Chemistry* sections—addresses the “Intervention Effects of Food-derived Polyphenols and Bioactive Peptides on Chronic Inflammation.” It compiles various studies that investigate how these bioactive compounds can modulate inflammation and enhance health outcomes. The collected manuscripts featured in this Research Topic cover a broad spectrum of topics, ranging from the molecular pathways influenced by specific polyphenols to the therapeutic applications of bioactive peptides in various disease contexts. One of the primary focuses is the modulation of oxidative stress and inflammatory pathways, which are key contributors to chronic inflammation.

## Intervention effects of food-derived polyphenols

For instance, one of the notable contributions is the study (Liu M. et al.) on dietary supplementation with mulberry leaf flavonoids and carnosic acid complex. This research delves into the synergistic effects of mulberry leaf flavonoids and carnosic acid in improving growth performance and antioxidant capacity in broilers. The findings reveal that the combination of these bioactive compounds enhances growth performance and antioxidant capacity in broilers by regulating the p38 MAPK/Nrf2 pathway. This research highlights the potential of these compounds as alternatives to antibiotics (particularly at a dosage of 150 mg/kg), promoting intestinal health and systemic antioxidant defenses.

In the context of neurodegenerative diseases, the review (Li S. et al.) titled *Neurodegenerative Diseases and Catechins: (–)-Epigallocatechin-3-gallate as a Modulator of Chronic Neuroinflammation and Oxidative Stress* delves into the neuroprotective effects of catechins, particularly the most abundant polyphenol in green tea, (-)-Epigallocatechin-3-gallate (EGCG). The discussion on how EGCG attenuates neuroinflammatory processes and oxidative stress mechanisms like scavenging free radicals, reducing oxidative stress and attenuating neuroinflammatory processes. Meanwhile, they underscore its potential as a therapeutic agent for conditions like Alzheimer's and Parkinson's diseases.

In a related review (Qi et al.) on *Zanthoxylum bungeanum* Maxim. (Chinese prickly ash) delves into the polyphenolic components of this traditional spice and their promising anti-inflammatory effects. The review synthesizes data from preclinical studies, suggesting that these polyphenols may offer therapeutic benefits for a range of inflammatory diseases, including ulcerative colitis, arthritis, and cardiovascular diseases. This comprehensive analysis not only highlights the potential of *Z. bungeanum* polyphenols as natural anti-inflammatory agents but also calls for further research to elucidate their mechanisms of action and therapeutic efficacy in humans.

While in the context of antimicrobial therapies, another review (Wang et al.) in this Research Topic addresses the use of polyphenolic natural products as photosensitizers in antimicrobial photodynamic therapy (aPDT). Given the rising concern of antibiotic resistance, this review is timely, offering a comprehensive overview of the potential for polyphenols like curcumin, quercetin, and resveratrol to serve as effective photosensitizers in aPDT. The review not only details the antimicrobial properties of these compounds but also explores their mechanisms of action, providing a solid foundation for future research aimed at developing novel, natural antimicrobial therapies.

Expanding on the topic of polyphenols, a significant contribution to this Research Topic is the study (Fu et al.) exploring the J-shaped association of dietary catechin intake with the prevalence of osteoarthritis (OA) in a large American cohort. This study presents intriguing evidence that moderate intake of specific catechins, such as epigallocatechin and EGCG, is associated with a reduced prevalence of OA, particularly when combined with physical activity. However, excessive intake of these catechins was identified as a risk factor, suggesting a nuanced relationship between catechin consumption and OA. This research emphasizes the importance of balanced dietary intake and highlights the potential for dietary interventions to mitigate OA risk.

Continuing the theme of anti-inflammatory properties, the review (Cozmin et al.) titled *Turmeric: From Spice to Cure. A review of the anti-cancer, radioprotective and anti-inflammatory effects of turmeric sourced compounds*. This review delves into the multifaceted pharmacological properties of turmeric, particularly curcumin, which has shown promise in cancer prevention and treatment, as well as in mitigating radiation-induced damage. The authors provide a thorough analysis of the molecular mechanisms by which curcumin exerts its effects, reinforcing the spice's potential as a cornerstone in integrative oncology and radiation therapy.

Moreover, the clinical trial (Karegar et al.) on ellagic acid supplementation in multiple sclerosis (MS) patients adds to the growing body of evidence supporting the anti-inflammatory and neuroprotective effects of polyphenols. The trial demonstrates that ellagic acid can significantly reduce inflammatory cytokines and modulate gene expression related to immune response, leading to improved clinical outcomes in MS patients. These results highlight the therapeutic potential of ellagic acid in managing chronic autoimmune diseases and call for further exploration in larger, more diverse patient populations.

In a similar context, the study (Janilkarn-Urena et al.) on dihydromyricetin (DHM) supplementation and its effects on ethanol-induced lipid accumulation and inflammation in a murine model of alcohol-associated liver disease (ALD) provides promising results. DHM, a bioactive polyphenol, was shown to reduce liver inflammation and improve lipid metabolism, suggesting its potential as a therapeutic agent for ALD. This study contributes valuable preclinical data supporting the use of DHM as a cost-effective and safe dietary supplement for managing ALD and other inflammatory liver conditions.

## Intervention effects of bioactive peptides

A review (Liu H. et al.) examines the application and mechanism of bioactive peptides (BAPs), focusing on their immunomodulatory properties. The review provides a comprehensive overview of how BAPs can regulate key signaling pathways such as MAPK and NF-κB, offering a natural alternative to non-steroidal anti-inflammatory drugs (NSAIDs) that avoids their associated adverse effects. This work emphasizes the potential of BAPs in managing chronic inflammation across various medical conditions.

In addition to BAPs, the review (Boboua et al.) titled *Valorization of Animal Waste Proteins for Agricultural Food Production and Medicinal Applications* addresses the sustainable utilization of animal waste proteins, transforming what is often considered a liability into a valuable resource. The review examines the potential of hydrolysates and peptides derived from animal waste proteins in various industries, including agriculture, food production, and medicine. The authors advocate for further research into the bioavailability and structure-activity relationships of these peptides to fully realize their therapeutic potential.

## Intervention effects of lifestyle

On the dietary front, a case study (Zhang et al) from Kashi Xinjiang investigated the relationship between the Dietary Inflammatory Index (DII) and metabolic syndrome (MS) among Uygur adults. This case study provides compelling evidence that dietary patterns can significantly influence the risk of developing MS and its components. The findings emphasize that a diet rich in anti-inflammatory foods, such as fruits and milk, correlates with a reduced prevalence of MS and hypertension, while a diet heavy in meat and eggs is associated with increased risks of high fasting glucose and obesity. These insights underscore the importance of dietary interventions in managing metabolic health, particularly in populations with distinct dietary habits.

Besides dietary habits, lifestyle choices can also increase oxidative stress, enhance mitogenic signaling pathways, and lead to genomic and epigenomic disturbances (4). Turning to lifestyle factors, a novel risk factor for knee osteoarthritis (KOA) is presented in a study (Huang et al.) examining the association between alcohol consumption, particularly pea-based alcoholic drinks, and the incidence of knee surgery in KOA patients. This study identifies a clear correlation between high alcohol intake and the increased risk of knee surgery, with pea-based alcoholic beverages emerging as a particularly potent risk factor. These findings offer new perspectives on dietary and lifestyle factors contributing to KOA progression and suggest potential avenues for patient education and prevention strategies.

## Antioxidant effect in modulating ROS homeostasis on plants

Finally, the research (Li N. et al.) on the crosstalk between melatonin and reactive oxygen species (ROS) in fruits and vegetables post-harvest preservation provides an important update on the role of melatonin in modulating ROS homeostasis. This review highlights the dual role of melatonin as both an antioxidant and a signaling molecule, suggesting new strategies for enhancing the post-harvest quality of fruits and vegetables through the application of melatonin.

## Conclusions

In conclusion, the studies presented in this Research Topic provide a comprehensive overview of the intervention effects of food-derived polyphenols and bioactive peptides on chronic inflammation. They not only advance our understanding of the molecular mechanisms involved but also pave the way for developing innovative dietary strategies and therapeutic interventions. We anticipate that this Research Topic will serve as a valuable resource for researchers and clinicians alike, driving further exploration and application of these bioactive compounds in promoting human health.

We extend our deepest appreciation to all the authors, reviewers, and editorial team members for their contributions to this Research Topic. Their efforts have been instrumental in advancing the field of nutritional immunology and expanding our knowledge of the health benefits of food-derived bioactive compounds.
